# A Large Mass over the Foot due to the Coexistence of an Eccrine Poroma and a Poroid Hidradenoma: A Case Report

**DOI:** 10.1055/s-0041-1732331

**Published:** 2021-10-01

**Authors:** Danilo Ryuko Cândido Nishikawa, Ana Caroline Leite da Silva, Marcos Yoshio Yano, Bruno Rodrigues de Miranda, Wu Tu Chung

**Affiliations:** 1 Departamento de Ortopedia, Cirurgia do Pé e Tornozelo, Hospital do Servidor Público Municipal de São Paulo, São Paulo, SP, Brasil

**Keywords:** eccrine poroma, foot, poroid hidradenoma, tumor

## Abstract

Eccrine poroma and poroid hidradenoma are uncommon benign poroid neoplasms derived from eccrine sweat glands. There are four types of poroid neoplasms according to the position within the skin layer: hidroacanthoma simplex, eccrine poroma, dermal duct tumor, and poroid hidradenoma. Poroid neoplasms usually arise as slow-growing solitary lesions and can present different clinical presentations, such as a foot mass, an ulceration lesion, a solid cyst, a bleeding lesion or suspected melanoma. Extremities are the most common sites, especially hands and feet. However, the coexistence of these two tumors in a single lesion is extremely rare. Surgical excision represents the main treatment and can be curative, preventing malignant changes and recurrence. We describe a rare solitary tumor over the foot with clinical and histopathological features of an association of an eccrine poroma and a poroid hidradenoma that was surgically treated with no recurrence at the midterm follow-up.

**Level of Evidence** IV, Case Report.

## Introduction

Eccrine poroma (EP) and poroid hidradenoma (PH) are rare benign poroid neoplasms (PNs) derived from eccrine sweat glands. 1 2 3 Usually, PNs arise as a solitary lesion with different clinical presentations, such as: foot mass, ulceration lesion, solid cyst, bleeding lesion or suspected melanoma. There are four types of variations of PN, classified according to the position within the skin layer: hidroacanthoma simplex, EP, dermal duct tumor, and PH. 4 Eccrine poroma involves the basal layer of the epidermis, while PH is confined to the dermis. 3 4 5

The literature about the coexistence of these two tumors in a single lesion is scarce. 5 6 7 Total resection followed by biopsy has been described as the main treatment, and it can be curative. 2 The present study aims to report a case with a large solitary mass over the foot originated by a rare association of EP and PH.

## Case Report

**Fig. 1:**
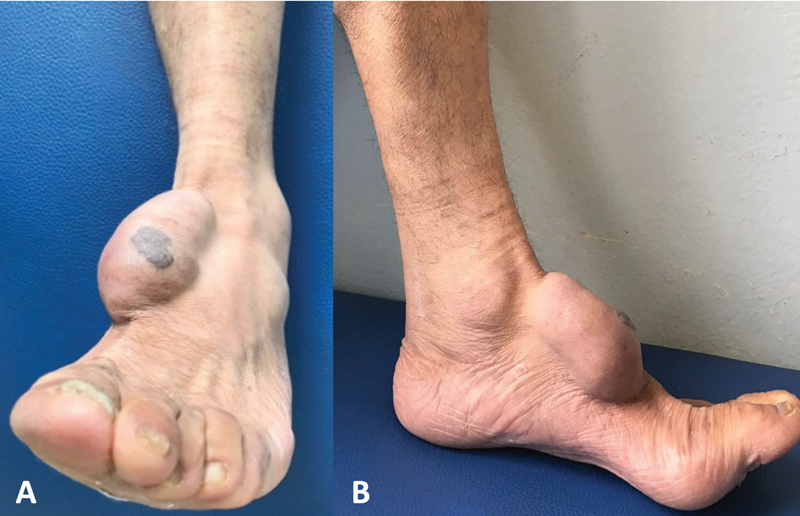
( **A** ) Frontal and ( **B** ) lateral clinical views of the large mass over the midfoot with well-defined edges and fibroelastic consistency, not adherent to the surrounding soft tissue. The slightly elevated black nodule on the top is the EP portion and most of the cystic tumor is the PH portion.

We present a 55-year-old male patient with a large, nontraumatic, painful mass in the left midfoot (∼ 7 × 4 cm) ( Fig. 1 ). He referred that it started as a hyperchromic nodular spot on the skin, and it grew slowly for the past 8 years. On the physical exam, the tumor presented well-defined edges, fibroelastic consistency, and it was not adherent to the surrounding soft tissue. The hyperchromic nodular spot measured ∼ 1.5 × 1 cm.

**Fig. 2:**
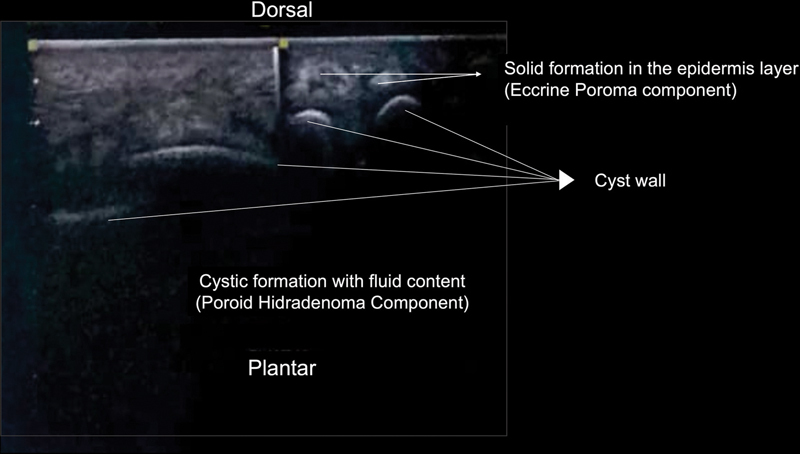
Ultrasound image showing a well-defined translucent cystic formation with a homogeneous fluid content within the dermis layer (PH component) and a solid formation in the epidermis layer (EP component).

An ultrasonography was taken to confirm its cystic form and to analyze its content. The result was a well-defined translucent cystic formation measuring 72 × 46 × 26mm with 3 mm of a homogeneous fluid content within the subcutaneous tissue ( Fig. 2 ). We suspected that the cystic tumor could be a common synovial cyst. However, we requested a dermatological evaluation due to the presence of a black spot on the skin over the cyst. Differential diagnosis was hypothesized, such as melanoma, pigmented basal cell carcinoma, and benign epithelial neoplasms.

**Fig. 3:**
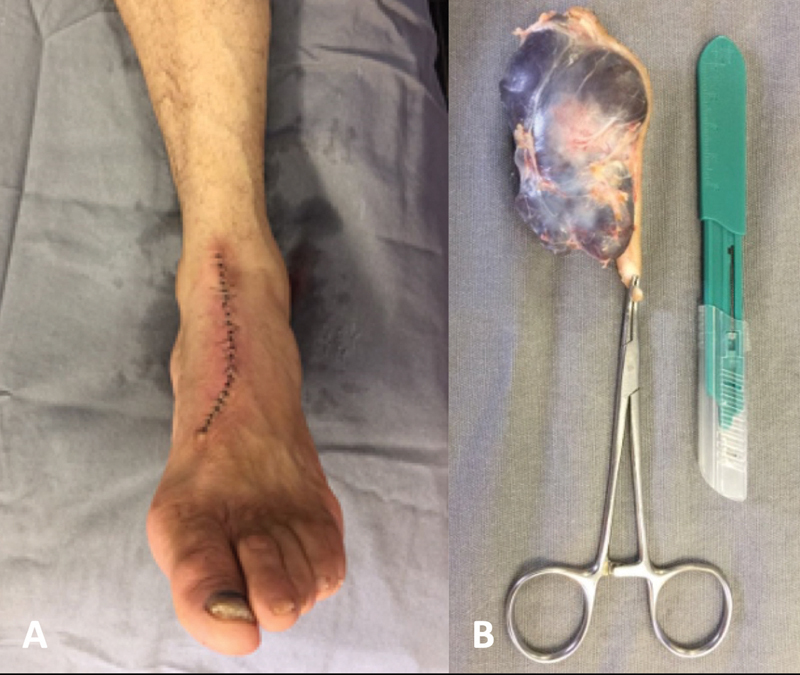
( **A** ) Dorsal approach and ( **B** ) the tumor totally excised. After resection, the cystic aspect of the lesion was observed.

We performed a total resection of the tumor, since its clinical aspect and ultrasound images presented benign features. A longitudinal dorsomedial approach centered over the tumor starting from the ankle joint to the distal third of the first metatarsal was performed, with the black spot being completely removed in an elliptical shape. Our decision for this approach was because it would allow us to dissect the tumor medially and laterally with less soft tissue manipulation, since the skin over cystic tumors tends to be thin ( Fig. 3 ). During dissection, care was taken to avoid damage to the nerve branch of the deep peroneal nerve, to the saphenous nerve and to the vein, as well as to the dorsalis pedis artery. We were able to dissect and excise the tumor entirely with clean margin ( Fig. 3 ). Then, the tumor was sent to the anatomopathological analysis.

**Fig. 4:**
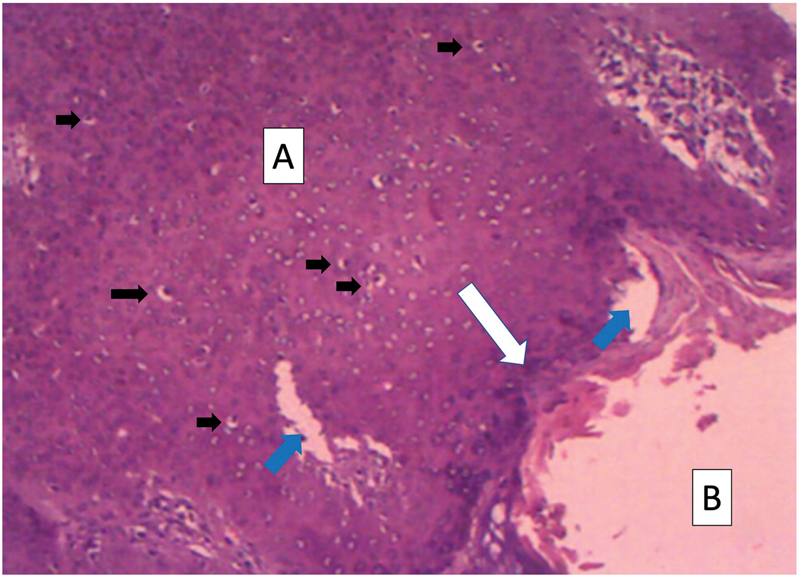
Histopathologic examination showing that the neoplasm consisted of both epidermal (Eccrine poroma) and dermal (Poroid Hidradenoma) components. The dark brown component ( **A** ) corresponds to the epidermis component of the neoplasm while the light one ( **B** ), to the dermis component. The epidermis component was full of small uniformly cuboid cells ( *black arrows* ), and some associated ductal spaces were also found within the tumor ( *blue arrows* ). The dermis component presented as a cystic tumor with fluid content surrounded by a solid wall ( *white arrow* ). The cyst wall consisted of layers of flattened poroid and cuticular cells. (Hematoxylin and Eosin 100x).

Histopathologic examination confirmed that the tumor was a PN consisting of both dermis (poroid hidradenoma) and epidermis (eccrine poroma) components. The majority of the neoplasm was from the dermis. This portion was characterized by cystic and solid parts. The solid part was the cyst wall and comprised one or two cell layers of poroid and cuticular cells with pink cytoplasm ( Fig. 4 ). The epidermis portion was characterized by a solid tumor, which consisted mainly of small, dark and monomorphous cuboid cells with scant cytoplasm and round nucleus, known as poroid cells ( Fig. 4 ). There were no histologic features of malignancy.

**Fig. 5:**
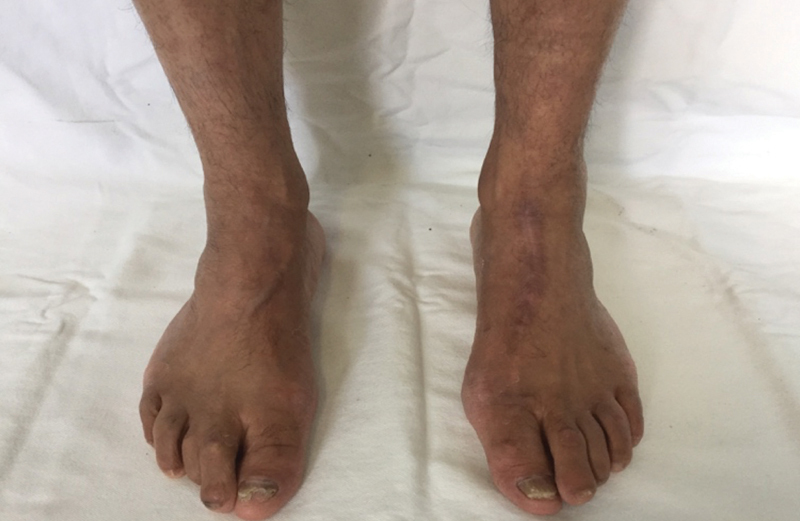
Clinical aspect of follow-up after 43 months of the excision of the tumor. There was no sign of recurrence of the mass in the dorsal of the left foot.

After 43 months of follow-up, the patient remains asymptomatic and fully active. He has returned to his previous activities without physical limitations. No clinical signs of recurrence have been observed so far ( Fig. 5 ).

## Discussion

The coexistence of EP and PH is rare, restricted to a few case reports in the knee and in the upper and lower back. 8 9 Nevertheless, they have demonstrated satisfactory clinical outcomes after complete excision of the tumor. No features of malignancy in the pathological analysis and no signs of recurrence were observed. The present study reports a unique case of a large solitary tumor on the foot consisting of an association of EP and PH that was successfully treated with surgical resection, with no signs of malignancy and recurrence. To our knowledge, only descriptions of separate presentations of these tumors on the feet have been published.

Eccrine poroma and PH share similar histological origin and cellular characteristics, such as monomorphic tumor cells, ductal differentiation and necrosis. 7 However, clinically, they present different aspects. Eccrine poroma is frequently a noncystic solid lesion with a nodular, granular, papillated aspect in various colors. Poroid hidradenoma presents as a solid and cystic tumor located entirely within the dermis. 6 Differently from EP, it does not occur in the palm or soles. 7 Our patient presented a solitary lesion with clinical features of EP and PH as a nodular hyperchromic spot and a cystic formation, respectively.

The etiology of these tumors is unknown. Inflammation and tissue regeneration might play a role, as there are descriptions of association with trauma, scarring, exposure to radiation, and immunosuppression. 7 However, the tumor of our patient has grown without any trauma, previous local infection, immunosuppressive diseases or history of exposure to radiation. In general, one of the etiology hypotheses of this association is probably because some parts of the skin and sweat glands may be induced to the tumorigenesis pathway at the same time. 5 Although rare, PE can evolve to an eccrine porocarcinoma (EPC). 2 10

The description of the diagnosis and treatment of this rare tumor may help orthopedic surgeons to look for skin tumors as differential diagnosis of tumors on the feet. We showed that surgical excision was an effective treatment with no recurrence of the tumor in the midterm follow-up.
