# Supplementary material for: A Large Mass over the Foot due to the Coexistence of an Eccrine Poroma and a Poroid Hidradenoma: A Case Report
Source: Rev Bras Ortop (Sao Paulo). 2021 Oct 1;59(Suppl 1):e5–8. doi: 10.1055/s-0041-1732331 (PMC11254418; doi:10.1055/s-0041-1732331)
Supplement: Related texts

# Research article: Poroma écrino associado a um hidradenoma poroide devido a coexistência de uma grande massa sobre o pé: Relato de caso

**Keywords:** hidradenoma poroide, pé, poroma écrino, tumor

## Abstract

O poroma écrino e o hidradenoma poroide são neoplasias poroides benignas raras, derivadas das glândulas sudoríparas écrinas. Existem quatro tipos de neoplasias poroides, conforme a posição na camada da pele, sendo denominadas hidroacantoma simples, poroma écrino, tumor do ducto dérmico e hidradenoma poroide. As neoplasias poroides geralmente surgem como uma lesão solitária, com crescimento lento, podendo apresentar diferentes apresentações clínicas, como massa sobre o pé, lesão ulcerada, cisto sólido, lesão hemorrágica ou suspeita de melanoma. As extremidades são os sítios mais comuns, especialmente as mãos e os pés. No entanto, a coexistência desses dois tumores em uma única lesão é extremamente rara. A excisão cirúrgica representa o principal tratamento, podendo ser curativa, prevenindo assim as alterações malignas e as recidivas. Descrevemos um raro tumor solitário no pé com características clínicas e histopatológicas de uma associação de poroma écrino e hidradenoma poroide tratado cirurgicamente sem recidiva no acompanhamento de médio prazo.

**Nível de Evidência** IV, Relato de Caso.

## Introdução

O poroma écrino (PE) e o hidradenoma poroide (HP) são neoplasias poroides (NPs) benignas raras derivadas das glândulas sudoríparas écrinas. 1 2 3 As NPs geralmente surgem como uma lesão solitária com diferentes manifestações clínicas, como massa sobre o pé, lesão ulcerada, cisto sólido, lesão hemorrágica ou suspeita de melanoma. Existem quatro tipos de variações de NP, classificadas em relação à posição dentro da camada da pele: hidroacantoma simples, PE, tumor do ducto dérmico e HP. 4 O PE envolve a camada basal da epiderme, enquanto o HP está confinado à derme. 3 4 5

A literatura sobre a coexistência desses dois tumores em uma única lesão é escassa. 5 6 7 A ressecção total seguida de biópsia tem sido descrita como o tratamento principal, e pode ser curativa. 2 O presente estudo tem como objetivo relatar um caso com uma grande massa solitária sobre o pé, originado a partir de uma rara associação de PE e HP.

## Relato de Caso

Apresentamos um paciente do sexo masculino de 55 anos com uma grande massa não traumática e dolorosa sobre o médio-pé esquerdo (∼ 7 × 4 cm) ( Fig. 1 ). Ele referiu que a lesão começou como uma mancha nodular hipercrômica na pele e cresceu lentamente nos últimos 8 anos. Ao exame físico, o tumor apresentava bordas bem definidas e consistência fibroelástica, não aderindo ao tecido mole circundante. A mancha nodular hipercrômica tinha o tamanho aproximado de 1,5 × 1cm.

**Fig. 1:** ( **A** ) Vista clínica frontal e ( **B** ) lateral de grande massa sobre o médio-pé, com bordas bem definidas, consistência fibroelástica e não aderente ao tecido mole circundante. O nódulo preto ligeiramente elevado na parte superior é a porção PE, e a maior parte do tumor cístico é a porção HP.

Foi realizada a ultrassonografia para confirmação de sua forma cística e análise do conteúdo. O resultado foi uma formação cística translúcida bem definida medindo 72 × 46 × 26 mm, com 3 mm de um conteúdo fluido homogêneo dentro do tecido subcutâneo ( Fig. 2 ). Havia a suspeita de que o tumor cístico poderia ser um cisto sinovial comum. Porém, solicitamos avaliação dermatológica, devido à presença de uma mancha preta na pele sobre o cisto. Cogitou-se a hipótese de que o diagnóstico diferencial seria melanoma, carcinoma basocelular pigmentado e neoplasias epiteliais benignas.

**Fig. 2:** Imagem de ultrassom mostrando uma formação cística translúcida bem definida, apresentando um conteúdo fluido homogêneo dentro da camada derme (componente HP) e uma formação sólida na camada epiderme (componente PE).

Foi realizada a ressecção total do tumor, já que o seu aspecto clínico e as imagens ultrassonográficas apresentavam características benignas. Foi realizada uma abordagem dorsomedial longitudinal centralizada sobre o tumor a partir da articulação do tornozelo até o terço distal do primeiro metatarso, sendo a mancha preta removida totalmente em forma de elipse. Nossa decisão por esta abordagem foi porque ela permitiria dissecar o tumor medial e lateralmente, com menos manipulação dos tecidos moles, já que a pele sobre os tumores císticos tende a ser fina ( Fig. 3 ). Durante a dissecção, foi tomado todo o cuidado para evitar danos ao ramo nervoso do nervo fibular profundo, ao nervo safeno e à veia, bem como à artéria dorsal do pé. Conseguimos dissecar e excisar totalmente o tumor com uma margem limpa ( Fig. 3 ). Em seguida, o tumor foi encaminhado para análise anatomopatológica.

**Fig. 3:** ( **A** ) Abordagem dorsal e ( **B** ) tumor totalmente excisado. Após a ressecção, observou-se o aspecto cístico da lesão.

O exame histopatológico confirmou que o tumor era uma NP, consistindo de ambos os componentes da derme (hidradenoma poroide) e da epiderme (poroma écrino). A maioria das neoplasias era da derme. Essa porção era caracterizada por partes císticas e sólidas. A parte sólida era a parede do cisto, abrangendo uma ou duas camadas de células poroides e cuticulares com citoplasma rosa ( Fig. 4 ). A porção da epiderme era caracterizada por um tumor sólido, consistindo principalmente de células cuboides pequenas, escuras e monomórficas, com citoplasma escasso e núcleo redondo, conhecidas como células poroides ( Fig. 4 ). Não havia características histológicas de malignidade.

**Fig. 4:** Exame histopatológico mostrando que a neoplasia era constituída por componentes epidérmicos (poroma écrino) e dérmicos (hidradenoma poroide). O componente marrom escuro ( **A** ) corresponde ao componente epiderme da neoplasia, enquanto o componente claro ( **B** ) corresponde ao componente derme. O componente da epiderme estava repleto de pequenas células uniformemente cuboides (setas pretas); também foram encontrados alguns espaços ductais relacionados dentro do tumor (setas azuis). O componente da derme apresentou-se como tumor cístico com conteúdo líquido circundado por uma parede sólida (seta branca). A parede do cisto era constituída por camadas de células poroides e cuticulares achatadas. (Hematoxilina-Eosina 100x).

Após 43 meses de acompanhamento, o paciente permanece assintomático e totalmente ativo. Ele voltou às suas atividades anteriores sem limitações físicas. Não foram observados sinais clínicos de recidiva até agora ( Fig. 5 ).

**Fig. 5:** Aspecto clínico do acompanhamento após 43 meses da excisão do tumor. Não havia sinais de recidiva da massa na região dorsal do pé esquerdo.

## Discussão

A coexistência de PE e HP é rara, restrita a poucos relatos de casos no joelho e nas partes superior e inferior das costas. 8 9 Porém, eles têm demonstrado evolução clínica satisfatória após a excisão completa do tumor. Não foi observada nenhuma característica de malignidade na análise patológica e nenhum sinal de recorrência. O presente estudo relata um caso único de grande tumor solitário sobre o pé, consistindo de uma associação de PE e HP, que foi tratado com sucesso através da ressecção cirúrgica, sem sinais de malignidade e recorrência. Até onde se tem conhecimento, apenas descrições de apresentações separadas desses tumores nos pés foram publicadas.

O PE e o HP compartilham a origem histológica, as características celulares semelhantes às células tumorais monomórficas, a diferenciação ductal e a necrose. 7 No entanto, clinicamente, apresentam aspectos diferentes. Frequentemente, o PE é uma lesão sólida não cística, com aspectos nodulares, granulares e papilados de várias cores. O HP se apresenta como um tumor sólido e cístico localizado inteiramente na derme. 6 Diferentemente do PE, o HP não ocorre na palma da mão ou na planta do pé. 7 Nosso paciente apresentava uma lesão solitária com características clínicas de PE e HP, nas formas de uma mancha nodular hipercrômica e de uma formação cística, respectivamente.

A etiologia desses tumores é desconhecida. A inflamação e a regeneração tissular podem ter grande importância, já que existem descrições de associação a traumas, cicatrizes, exposição à radiação e imunossupressão. 7 No entanto, o tumor do nosso paciente cresceu sem qualquer trauma, infecção local anterior, doenças imunossupressoras ou histórico de exposição à radiação. Em geral, uma das hipóteses etiológicas dessa associação é porque, provavelmente, algumas partes da pele e das glândulas sudoríparas podem ser induzidas à via da tumorigênese ao mesmo tempo. 5 Embora raro, o PE pode evoluir para um porocarcinoma écrino (PCE). 2 10

A descrição do diagnóstico e do tratamento desse tumor raro pode ajudar o cirurgião ortopédico na busca por tumores cutâneos como um diagnóstico diferencial de tumores nos pés. Ficou demonstrado que a excisão cirúrgica foi um tratamento eficaz, sem recorrência do tumor no acompanhamento de médio prazo.
